# The chloroplast genome of the *Iris japonica* Thunberg (Butterfly flower) reveals the genomic and evolutionary characteristics of *Iris* species

**DOI:** 10.1080/23802359.2022.2118000

**Published:** 2022-10-11

**Authors:** Xinyi Zhang, Heyu Yang, Bin Wu, Haimei Chen

**Affiliations:** Institute of Medicinal Plant Development, Chinese Academy of Medical Sciences and Peking Union Medical College, Beijing, P.R. China

**Keywords:** *Iris*, chloroplast genome, hypervariable region, selective pressure analysis, phylogenetic analysis

## Abstract

*Iris japonica* Thunberg is one of the horticultural species belonging to the *Iris* genus and Iridaceae family. Previous studies have revealed its hepatoprotective activity and ornamental values. However, little genetic and genomic information about this species is available. Here, to decipher the chloroplast genome and reveal its evolutionary characteristics, we sequenced, *de novo* assembled, and comprehensively analyzed the chloroplast genome of *I. japonica*. The genome was 152,453 bp in length and displayed a circular structure with a large single-copy region, a small single-copy region, and two inverted repeat regions. It contained 131 genes, including 85 protein-coding genes, eight ribosomal RNA genes, and 38 transfer RNA genes. We also identified 23 microsatellite repeat sequences, 34 tandem repeat sequences, and 60 dispersed repeat sequences in the chloroplast genome of *I. japonica*. Sequence divergence analyses of the chloroplast genomes of 20 *Iris* species revealed that the top four most highly variable regions were *ndhC-trnV-UAC*, *rpl22-rps19*, *rps16-trnQ-UUG*, and *trnG-UCC-trnR-UCU*. Phylogenetic analysis showed that *I. japonica* was most closely related to *I. tectorum*. This study reported a new chloroplast genome of *I. japonica* and performed comparative analyses of 20 *Iris* chloroplast genomes. The results would facilitate the evolutionary research and development of molecular markers for *Iris* species.

## 1. Introduction

The butterfly flower (*Iris japonica* Thunberg) belongs to the Iridaceae family and is native to Japan and China. The butterfly flower has ornamental value for its beautiful butterfly-shaped flowers called ‘Hudie Hua’ in China. Moreover, *I. japonica* is a medicinal plant for treating bronchitis, internal injuries, rheumatism, and swelling. The butterfly flower has many biological activities, such as antioxidant, anti-mutagenic, anti-angiogenic, anti-inflammatory, and hypoglycemic functions (Xu et al. 2021). Although many studies about the medicinal value of *I. japonica* have been conducted, no work has studied its genetic and genomic information, limiting the species identification and evolutionary analysis of *I. japonica*.

Complete chloroplast genomes are circular, linear, or polycyclic double-stranded DNA molecules, with a length mostly around 120–160 kb (Bock 2015). The complete chloroplast genome sequence can be used for the development of DNA barcodes, the determination of phylogenetic relationships, and the identification of patterns of gene loss and adaptive changes that optimize photosynthesis (Olejniczak et al. 2016).

Recently, many chloroplast genomes have been deciphered due to the advanced genome sequencing technologies and bioinformatics tools. One study sequenced and assembled the chloroplast genomes of 14 Korean-native *Iris* species (Kang et al. 2020). The 14 *Iris* chloroplast genomes were compared, and Bayesian phylogenetic trees were constructed. Five other studies reported the sequencing and assembly of the chloroplast genomes of *Iris sanguinea* (Lee et al. 2017), *Iris domestica* (Ai et al. 2019), *Iris loczyi* (Choi et al. 2020), *Iris tectorum* (Liu et al. 2020), and *Iris lactea* var. *chinensis* (Cai et al. 2021). Additionally, one assembly of the chloroplast genome of *I. japonica* was available in Genbank (NC_060499.1). However, a detailed analysis of this assembly was not reported.

Here, we sequenced and assembled the chloroplast genome of *I. japonica* and conducted a comparative analysis with 19 other *Iris* species. Our study presented the very first detailed analysis of the *I. japonica* chloroplast genome. The results will lay a solid foundation for the future development of *I. japonica*-based medicine.

## 2. Materials and methods

### 2.1. Plant materials and DNA extraction

We collected the fresh leaves of the *I. japonica* plants from Guangxi Province, China (E104°26′, N20°54′). The voucher specimen (IMPLADS2021009040) and its DNA sample (IMPLADS2021009040_dna01) were deposited at the Herbarium of Institute of Medicinal Plant Development (IMD), China (http://www.implad.ac.cn/, Haimei Chen, e-mail: hmchen@implad.ac.cn). We extracted the total DNA of one *I. japonica* sample using the plant genomic DNA kit (Tiangen Biotech, Beijing, Co., Ltd.). Total DNA was separated with electrophoresis in 1.2% agarose gels and analyzed with the Nanodrop 2000 spectrophotometer (Thermo Fisher Scientific Inc., Waltham, MA, USA) to assess quality and purity. Our study, including sample collection, was conducted in compliance with relevant institutional, national, and international guidelines and laws.

### 2.2. Plastome sequencing, assembly, and annotation

The 1 μg total DNA of *I. japonica* was used to construct the sequencing libraries using the TruSeq DNA Sample Prep Kit (Illumina, Inc., San Diego, CA, USA) following the manufacturer’s instructions and the libraries were sequenced on the Illumina NovaSeq PE150 instrument (Illumina Inc., San Diego, CA, USA) (Caporaso et al. 2012).

The genome was *de novo* assembled using NOVOPlasty (version 3.8.3) with the default parameters (Dierckxsens et al. 2017). The chloroplast genome of *I. japonica* was annotated with CPGAVAS2 (version 2.0) web service (Shi et al. 2019). The circular gene map of the chloroplast genome was drawn by the cpgavas2 web server (http://47.96.249.172:16019/analyzer/view). The raw data, final genome assembly sequence, and annotated information were deposited in CNCB-NGDC, with the accession numbers SRR18908135 and GWHBISG01000000.

### 2.3. Comparative genome analysis

The complete chloroplast genome sequences of 20 *Iris* species were downloaded from the GenBank database (Supplementary Material, Table S1), and the *I. japonica* chloroplast genome was obtained in this study (GWHBISG01000000). We conducted the comparative analysis of these 20 genomes using mVISTA with the ShuffleLAGAN mode (Brudno et al. 2003). The genes around the border of the IR, LSC, and SSC regions of the 21 *Iris* species were analyzed using IRscope online software (Amiryousefi et al. 2018) (https://irscope.shinyapps.io/irapp/). The genetic distance of the intergenic spacer (IGS) was calculated using the distmat program from EMBOSS (v6.3.1) with the Kimura 2-parameter (K2P) evolutionary model (Rice et al. 2000). We also compared our new chloroplast genome with the one deposited in the Genebank (NC_060499) using SeqMan software (version7.1.0).

### 2.4. Selective pressure analysis of protein-coding genes

The selective pressure of the protein-coding genes (PCGs) in the chloroplast genome was analyzed based on nucleotide substitution rate variation (Song et al. 2020). To detect the genes of the *I*. *japonica* chloroplast genome that were under selection, we extracted 71 common PCGs among the 20 *Iris* chloroplast genomes, performed multiple sequence alignment using MAFFT (v7.313), and constructed a maximum likelihood (ML) tree using IQTREE (v1.6.10) (Nguyen et al. 2015). The selective pressure of PCGs was analyzed using the aBSREL (adaptive branch-site random effects likelihood) model implemented in Hyphy software (v2.2.4) (Smith et al. 2015).

### 2.5. Phylogenetic analysis

Phylogenetic analysis was performed using the chloroplast genomes of 33 *Iris* species, and *Crocus cartwrightianus (NC_041459)* was selected as the outgroup. We used the PhyloSuite (v1.1.16) (Zhang et al. 2020) to extract the coding sequences of 66 common PCGs (*atpA*, *atpB*, *atpE*, *atpF*, *atpH*, *ccsA*, *cemA*, *infA*, *matK*, *nadA*, *ndhB*, *ndhC*, *ndhE*, *ndhF*, *ndhG*, *ndhH*, *ndhJ*, *ndhK*, *petA*, *petB*, *pe*t*D*, *petG*, *petL*, *petN*, *ps*a*A*, *psaB*, *psaC*, *psaJ*, *psbA*, *psbC*, *psbD*, *psbE*, *psbF*, *psbH*, *psbI*, *psbJ*, *psbK*, *psbL*, *psbM*, *psbT*, *rbcL*, *rpl1*4, *rpl*16, *rpl*20, *rpl*22, *rpl*23, *rpl*32, *rpl*33, *rpl*36, *rpoA*, *rpoB*, *rpoC*1, *rpoC*2, *rps*2, *rps*3, *rps*4, *rps*7, *rps*8, *rps*11, *rps*12, *rps*14, *rps*15, *rps*16, *rps*18, *ycf*1 and *ycf*2) of the chloroplast genome of these 34 species. Then the coding sequences of the 66 shared PCGs were aligned using MAFFT (v7.313) (Katoh et al. 2002). The phylogenetic tree was constructed using the ML method implemented in IQ-TREE (v1.6.8) with the TVM + F + I + G4 nucleotide substitution model (Smith et al. 2015). Finally, we assessed the reliability of the phylogenetic tree by the bootstrap test with 1000 replications and visualized the final tree using iTOL (https://itol.embl.de/) (Letunic and Bork 2019).

## 3. Results

### 3.1. Genome organization and compositions

**Figure 1.**
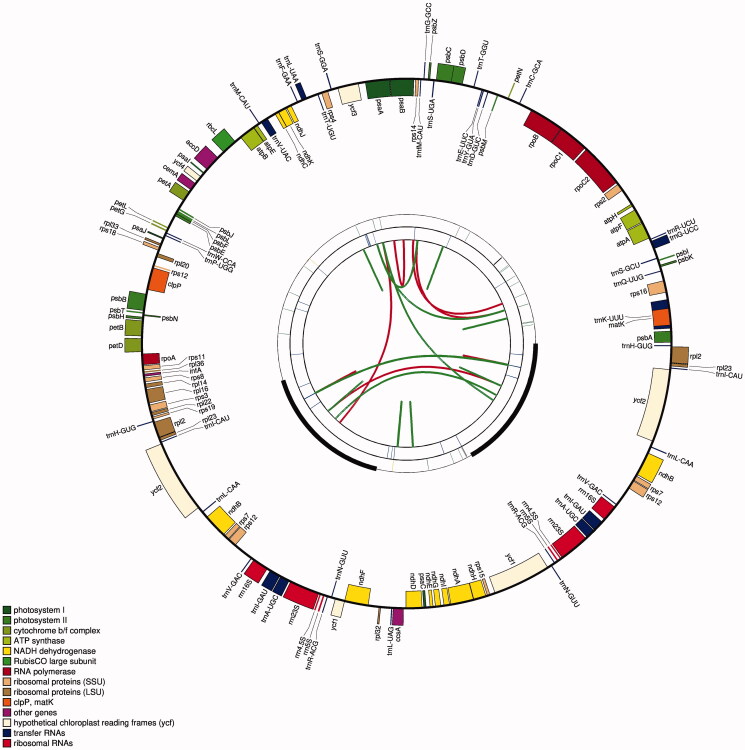
Graphic representation of features identified in *I. japonica* chloroplast genome using CPGAVAS2 (http://47.96.249.172:16019/analyzer/view). The map contains four circles. From the center going outward, the first circle shows the distributed repeats connected with red (the forward direction) and green (the reverse direction) arcs. The next circle shows the tandem repeats marked with short bars. The third circle shows the LSC, SSC, IRa, and IRb regions. The microsatellite sequences are shown as short bars on the circle. The fourth circle shows the genes having different colors based on the functional groups. The genes of the outside of the circle are transcribed clockwise. And the genes on the inside of the circle are transcribed anticlockwise. The functional classification is shown at the bottom left.

The chloroplast genome of *I. japonica* presented a typical circular DNA molecule with a total length of 152,453 bp (Figure 1). It had a conserved tetrapartite structure, including an LSC region, an SSC region, and a pair of IR regions, with lengths of 83,252, 18,489, and 25,356 bp, respectively. The total GC content in the chloroplast genome of *I. japonica* was 37.84%. The GC content of the IR region (43.03%) was higher than those of the SSC (31.38%) and LSC (36.12%) regions.

131 genes, including 85 PCGs, 38 tRNA genes, and eight rRNA genes, which consisted of two copies of *rrn*16S, *rrn*23S, *rrn*4.5S, and *rrn*5S genes were identified in the chloroplast genome of *I. japonica*. The gene structures of 21 *cis*-splicing genes are shown in Figure S1 (Supplementary Material). The 21 *cis*-splicing genes contained 13 PCGs and eight tRNA genes. All the *cis*-splicing genes had only one intron each except *ycf*3 and *clp*P, which contained two introns (Supplementary Material, Table S2).

### 3.2. Comparative analysis of 20 Iris chloroplast genomes

**Figure 2.**
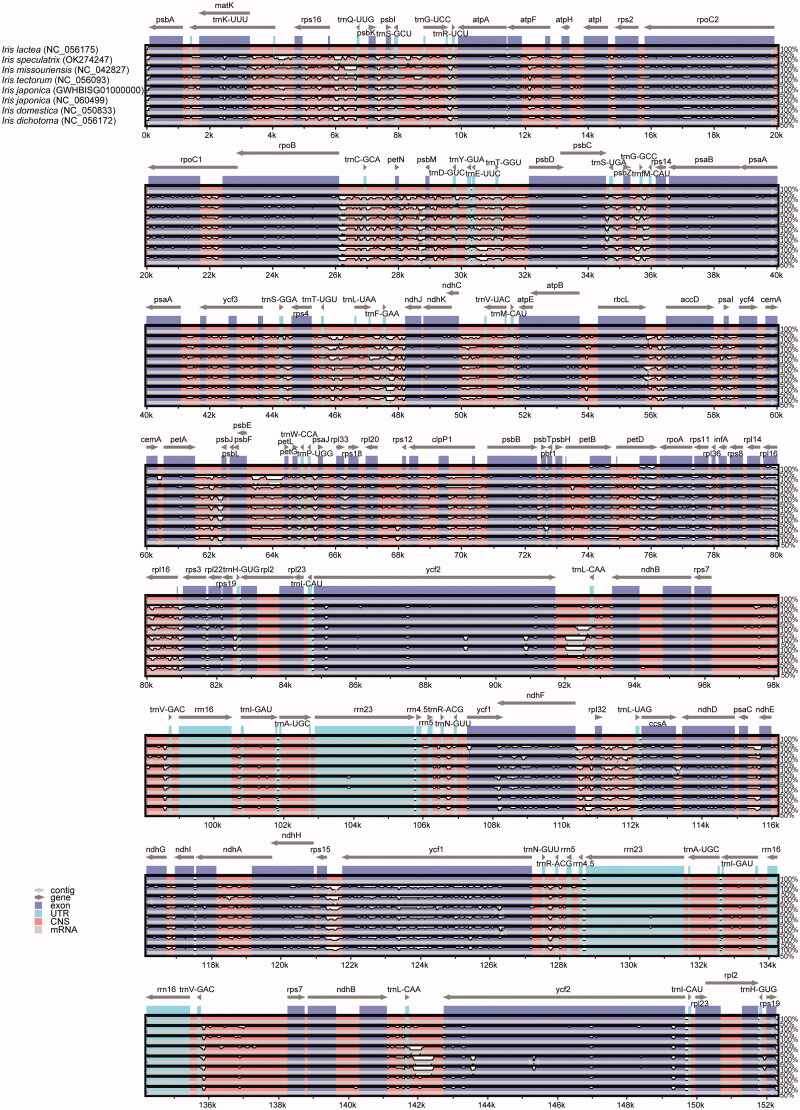
Sequence alignment of seven chloroplast genome of *Iris* species using mVISTA and chloroplast genome of *I. lactea* (NC_056175) as reference. The top arrow shows transcription direction, blue color indicates protein-coding regions, pink color shows non-coding sequences and light green indicates tRNAs and rRNAs. The x-axis represents the coordinates in the cp genome while y- axis represents percentage identity within 50–100%.

The chloroplast genome sequences of 21 species including the 19 species listed in Table S1 (Supplementary Material), *I. japonica* (GWHBISG01000000) obtained in this study, and *I. japonica* (NC_060499) were aligned using mVISTA to identify the variations in nucleotide sequence. The results showed that some highly variable regions were detected, including the protein-coding region of *ycf1*, *petB*, and *petD* and the IGS regions of *rps16-trnQ-UUG*, *rpoB-trnC-GCA*, *ndhC-trnV-UAC*, *rpl33-rps18*, and *rpl22-rps19* (Figure 2). The alignment of the chloroplast genomes of the 14 other species is shown in Figures S2 and S3 (Supplementary Material).

### 3.3. Ir structure analysis of 20 Iris species

**Figure 3.**
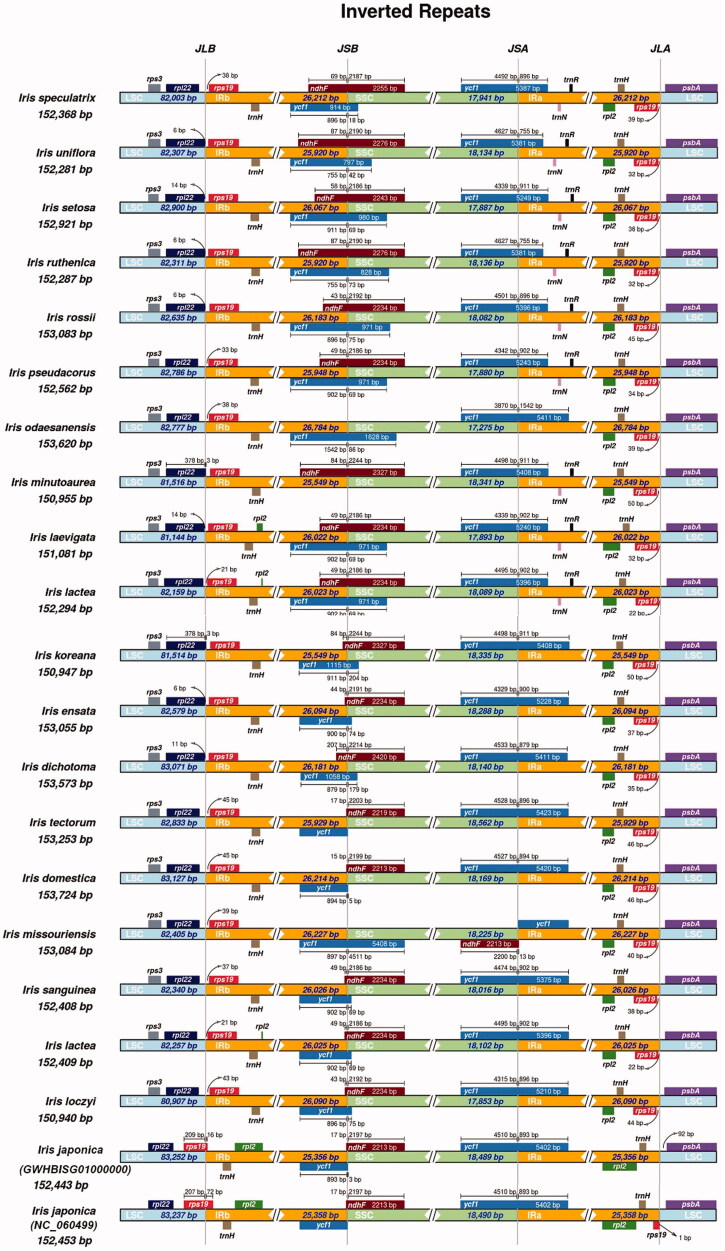
Comparison of LSC, SSC, and IR boundary areas of 21 *Iris* species.

IR boundary analysis of 21 complete chloroplast genomes of *Iris* species showed that most had the same boundary structure (Figure 3). For the 20 species except for *I. japonica*, both *rpl22* and *rps*19 genes were located around the border area of the LSC and IRb regions. By contrast, the *rps*19 gene of *I. japonica* (GWHBISG01000000) was present in the LSC region. Besides, the *ndhF* and *ycf1* genes of *I. tectorum*, *I. japonica*, and *I. domestica* were located at the border area of IRb and SSC. The *ycf*1 gene of most species spanned the SSC and IRb regions. The *psbA* genes were found at the border area of LSC and IRa.

### 3.4. Hypervariable region identification and selective pressure analysis

To find highly variable regions among the 20 *Iris* chloroplast genomes, we calculated the genetic distance of IGS regions using the K2P model. K2P values of *ndhC-trnV-UAC*, *rpl22-rps19*, *rps16-trnQ-UUG*, *trnG-UCC-trnR-UCU*, *rpl36-infA*, *trnC-GCA-petN*, *rpoB-trnC-GCA*, *rpl33-rps18*, *rps19-trnH-GUG*, *trnH-GUG-rpl2*, *rpl2-trnH-GUG*, and *psbK-psbI* were high at 27.05, 19.79, 15.97, 11.69, 10.45, 8.25, 7.95, 6.42, 6.35, 6.12, 6.12, and 6.05, respectively (Supplementary Material, Figure S4). The variations in these IGS regions were large, which suggested that these regions could be used to develop potential molecular markers for species discrimination of *Iris* species.

Using the aBSREL model, we found that the genes *rpoC2* and *ycf1* were positively selected in *I. japonica*. Moreover, the genes *ycf2*, *ndhG*, and *rpl20* were positively selected in other *Iris* species. These genes may be related to the adaptation of these *Iris* species to diverse environmental conditions (Supplementary Material, Table S3).

### 3.5. Phylogenetic analysis

**Figure 4.**
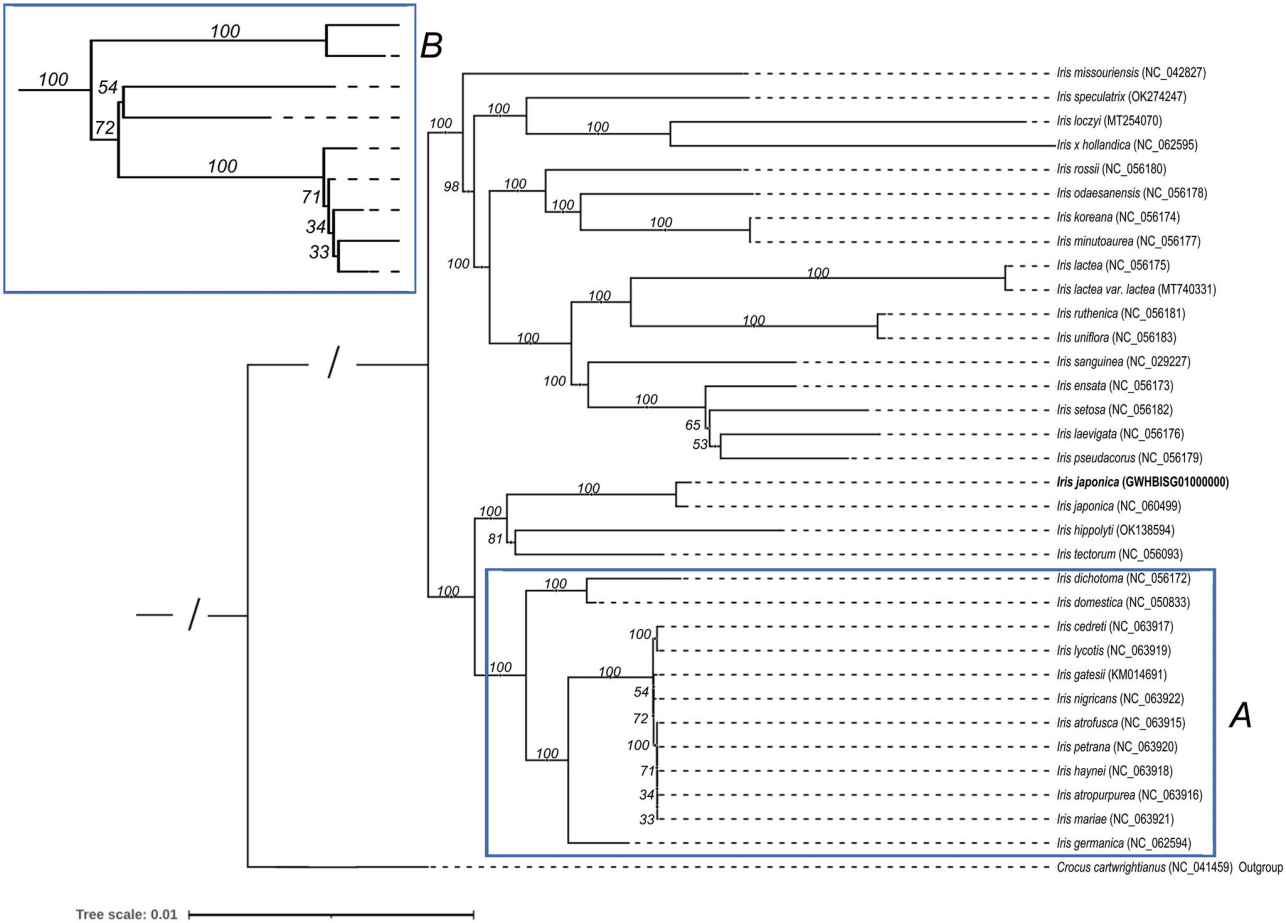
The phylogenetic tree of 21 *Iris* species. *Crocus cartwrightianus* was selected as the outgroup. The tree was constructed using the maximum-likelihood method based on the concatenated sequences from 66 shared proteins. Bootstrap support values were calculated from 1000 replicates. B box is a magnification of A box.

In this study, the chloroplast genome sequence of *I. japonica* (GWHBISG01000000) was aligned with 32 previously published genome sequences. The 32 published genome sequences are the chloroplast genome sequences of *I. sanguinea* (Lee et al. 2017), *I. missouriensis* (Joyce et al. 2018), *I. speculatrix* (Kang et al. 2020), *I. loczyi* (Choi et al. 2020), *I. x hollandica*, *I. rossii* (Kang et al. 2020), *I. odaesanensis* (Kang et al. 2020), *I. koreana* (Kang et al. 2020), *I. minutoaurea* (Kang et al. 2020), *I. lactea* (Kang et al. 2020), *I. ruthenica* (Kang et al. 2020), *I. uniflora* (Kang et al. 2020), *I. ensata* (Kang et al. 2020), *I. setosa* (Kang et al. 2020), *I. hippolyti*, *I. tectorum*, *I. dichotoma* (Kang et al. 2020), *I. laevigata* (Kang et al. 2020) and *I. pseudacorus* (Kang et al. 2020), *I. lactea var lactea* (Cai et al. 2021), *I. japonica* (NC_060499), *I. domestica*, *I. cedreti* (Volis et al. 2022), *I. lycotis* (Volis et al. 2022), *I. gatesii*, *I. nigricans* (Volis et al. 2022), *I. atrofusca* (Volis et al. 2022), *I. petrana* (Volis et al. 2022), *I. haynei* (Volis et al. 2022), *I. atropurpurea* (Volis et al. 2022), *I. mariae* (Volis et al. 2022) and *I. germanica*. Moreover, *I. japonicum* was sister to moderately-supported clade containing *I. tectorum* and *I. hippolyti*. (Figure 4). During this analysis, we found that another sequence from the same species was released in GenBank (NC_060499.1). Phylogenetic analysis suggested that the two sequences were highly similar. Sequence comparison showed 254 variations between the two sequences (Supplementary Material, Table S4).

## 4. Discussion

In this study, we sequenced the chloroplast genome of *I. japonica* and conducted comparative and evolutionary analyses of the chloroplast genomes with 19 other *Iris* chloroplast genomes. Overall, the *I. japonica* chloroplast genome was similar to those of the 19 other *Iris* species.

Notably, comparative analysis found some variations in genome organization. Different gene contents were noted at the border areas of the IR and LSC/SSC regions among the 21 *Iris* species. The *rps19* of only one species was present in the LSC region. However, the duplicated *rps19* of other 20 species were located in the IRa and IRb regions. This type of junction based on the different positions of *rps*19 was also found in the chloroplast genomes of *Aphelandra knappiae* and *Blepharis ciliaris*, which caused by the contraction and expansion in the IR regions (Alzahrani et al. 2020).

Highly variable regions in the chloroplast genome can serve as the DNA barcodes for evaluating phylogeny (Dong et al. 2012) and taxa classification (Dong et al. 2014). On the basis of genetic distances, some highly variable regions were observed in the 20 *Iris* chloroplast genomes, including *ndhC-trnV-UAC*, *rpl22-rps19*, *rps16-trnQ-UUG*, *trnG-UCC-trnR-UCU*, *rpl36-infA, and trnC-GCA-petN*. In the future, we will test the possibility of distinguishing these *Iris* species using molecular markers developed from these regions.

As described previously, only one comprehensive analysis of the phylogenetic relationships was reported. In that study, 14 Korean *Iris* species were separated into four clades (Kang et al. 2020). Here, we conducted phylogenetic analysis using 20 *Iris* species, including the14 reported species. Our study included a systematic analysis with six additional *Iris* chloroplast genomes. In our analysis, the 14 species reported previously were also clustered into four clades. For the newly added five species, the sister relationships between *I. loczyi* and *I. speculatrix*, and between *I. tectorum* and *I. japonica* were observed. In summary, the results obtained from this study will help us unveil the chloroplast genome evolution of *Iris* species.

## Supplementary Material

Supplemental Material

## Data Availability

The sample was stored at the Herbarium of the Institute of Medicinal Plant Development, Beijing, China, with the voucher numbers Implad201808209. The chloroplast genome sequence and the related annotations were available at https://ngdc.cncb.ac.cn/gwh in the Genome Warehouse in China National Genomics Data Center [38] with accession number GWHBISG01000000. The raw sequencing data for the Illumina platforms were deposited in CNCB-NGDC (https://ngdc.cncb.ac.cn/?lang=en). The accession numbers of Bioproject, Biosample, and GSA are PRJCA009243, SAMC781189, and CRA007000, respectively.
