# Supplementary material for: The chloroplast genome of the Iris japonica Thunberg (Butterfly flower) reveals the genomic and evolutionary characteristics of Iris species
Source: Mitochondrial DNA B Resour. 2022 Oct 11;7(10):1776–82. doi: 10.1080/23802359.2022.2118000 (PMC9559474; doi:10.1080/23802359.2022.2118000)
Supplement: Supplemental Material [file TMDN_A_2118000_SM2080.docx]

**The chloroplast genome of the butterfly flower (*Iris*** ***japonica* Thunb.) reveals the genomic and evolutionary characteristics of *Iris* species**

**Authors:**

Xinyi Zhang**^#^**, Heyu Yang**^#^**, Bin Wu*, Haimei Chen*

**Author affiliation:**

^1^Institute of Medicinal Plant Development, Chinese Academy of Medical Sciences and Peking Union Medical College, Beijing 100193, P. R. China.

**^#^Contributed equally to this work**

**^*^Corresponding Author:**

Haimei Chen: No. 151, Malianwa North Road, Haidian District, Beijing 100093, P.R. China. Email address: hmchen@implad.ac.cn; Phone: +86-10-57833111; Fax: +86-10-62899715;

Bin Wu: No. 151, Malianwa North Road, Haidian District, Beijing 100093, P.R. China. Email address: bwu@implad.ac.cn; Phone: +86-10-57833201; Fax: +86-10-62899715;

**E-mails:**

Xinyi Zhang: [473353782@qq.com](mailto:473353782@qq.com)

Heyu Yang: [18700944808@163.com](mailto:18700944808@163.com)

Bin Wu: [bwu@implad.ac.cn](mailto:bwu@implad.ac.cn)

Haimei Chen: hmchen@implad.ac.cn

# Table S1. The list of the nineteen *Iris* chloroplast genome used in our study.

| Name of species | Accession number of species |
| --- | --- |
| *Iris lactea* | NC_056175 |
| *Iris lactea* var *lactea* | MT740331 |
| *Iris ruthenica* | NC_056181 |
| *Iris uniflora* | NC_056183 |
| *Iris laevigata* | NC_056176 |
| *Iris pseudacorus* | NC_056179 |
| *Iris setosa* | NC_056182 |
| *Iris ensata* | NC_056173 |
| *Iris sanguinea* | NC_029227 |
| *Iris koreana* | NC_056174 |
| *Iris minutoaurea* | NC_056177 |
| *Iris odaesanensis* | NC_056178 |
| *Iris rossii* | NC_056180 |
| *Iris loczyi* | MT254070 |
| *Iris speculatrix* | OK274247 |
| *Iris missouriensis* | NC_042827 |
| *Iris tectorum* | NC_056093 |
| *Iris domestica* | NC_050833 |
| *Iris dichotoma* | NC_056172 |

# Table S2. Gene contents of *I. japonica* chloroplast genome

| Category | Group of genes | Name of genes |
| --- | --- | --- |
| rRNA | rRNA genes | *rrn*23S（×2）*,rrn*16S（×2）*,rrn*5S（×2）*,rrn*4.5S（×2） |
| tRNA | tRNA genes | 38 trn genes（8 contain an intron） |
| Self replication | Large subunit of ribosome | *rpl*14, *rpl*16, *rpl*2, *rpl*2, *rpl*20, *rpl*22, *rpl*23, *rpl*23, *rpl*32, *rpl*33, *rpl*36 |
|  | DNA dependent RNA polymerase | *rpo*A, *rpo*B, *rpo*C1, r*po*C2 |
|  | Small subunit of ribosome | *rps*11, *rps*12, *rps*12, *rps*14, *rps*15, *rps*16, *rps*18, *rps*19, *rps*2, *rps*3, *rps*4, *rps*7, *rps*7, *rps*8 |
| Photosynthesis | Subunits of ATP synthase | *atp*A, *atp*B, *atp*E, *atp*F, *atp*H |
|  | Subunits of photosystem II | *psb*A, *psb*B, *psb*C, *psb*D, *psb*E, *psb*F, *psb*I, *psb*J, *psb*K, *psb*L, *psb*M, *psb*N, *psb*T, *psb*Z, *ycf*3 |
|  | Subunits of NADH-dehydrogenase | *ndh*A, *ndh*B, *ndh*B, *ndh*C, *ndh*D, *ndh*E, *ndh*F, *ndh*G, *ndh*H, *ndh*I, *ndh*J, *ndh*K |
|  | Subunits of cytochrome b/f complex | *pet*A, *pet*B, *pet*D, *pet*G, *pet*L, *pet*N |
|  | Subunits of photosystem I | *psa*A, *psa*B, *psa*C, *psa*I, *psa*J |
|  | Subunit of rubisco | *rbc*L |
| Other genes | Subunit of Acetyl-CoA-carboxylase | *acc*D |
|  | c-type cytochrom synthesis gene | *ccs*A |
|  | Envelop membrane protein | *cem*A |
|  | Protease | *clp*P |
|  | Translational initiation factor | *inf*A |
|  | Maturase | *mat*K |
| Genes of unknown functions Open Reading | | *ycf4,ycf1* （×2）*,ycf2* （×2） |

# Table S3. Selection pressure analysis of six genes in the chloroplast genome of *Iris* species

| Gene | Species | Optimized branch length | LRT | p-value |
| --- | --- | --- | --- | --- |
| *acc*D | *Iris odaesanesis*  *Iris missouriensis*  *Iris speculatrix* | 0.0108 | 41.0067 | 0.0000 |
|  |  | 0.0114 | 23.9444 | 0.0001 |
|  |  | 0.0118 | 21.7693 | 0.0002 |
| *ndh*G | *Iris tectorum* | 0.0101 | 84.1795 | 0.0000 |
| *rpl*20 | *Iris loczyi* | 0.0280 | 34.9127 | 0.0000 |
| *rpo*C2 | *Iris tectorum*  *Iris ensata*  *Iris odaesanesis*  *Iris loczyi*  *Iris japonica*  *Iris rossii* | 0.0101 | 146.4790 | 0.0000 |
|  |  | 0.0063 | 138.3986 | 0.0000 |
|  |  | 0.0108 | 67.2728 | 0.0000 |
|  |  | 0.0280 | 22.2354 | 0.0002 |
|  |  | 0.0092 | 20.1852 | 0.0004 |
|  |  | 0.0092 | 12.0623 | 0.0255 |
| *ycf*1 | *Iris japonica*  *Iris odaesanesis*  *Iris loczyi*  *Iris tectorum* | 0.0092 | 60.0414 | 0.0000 |
|  |  | 0.0108 | 25.4869 | 0.0000 |
|  |  | 0.0280 | 23.1656 | 0.0001 |
|  |  | 0.0101 | 10.8432 | 0.0488 |
| *ycf*2 | *Iris loczyi*  *Iris tectorum* | 0.0280 | 74.3844 | 0.0000 |
|  |  | 0.0101 | 42.1975 | 0.0000 |

# Table S4. Comparison of the *I. japonica* chloroplast genome sequenced in this study (GWHBISG01000000) and the one released (NC_060499.1).

| **GWHBISG01000000 NC_060499.1** | | | |
| --- | --- | --- | --- |
| **Position** | **Sequence** | **Position** | **Sequence** |
| 72 | G | 65 | A |
| 246 | G | 239 | A |
| 2114 | A | 2107 | C |
| 3586 | T | 3579 | - |
| 4109 | A | 4101 | - |
| 4225 | C | 4216 | T |
| 4232 | T | 4223 | - |
| 5064 | G | 5054 | T |
| 5485 | G | 5475 | T |
| 6028 | A | 6018 | G |
| 6262 | - | 6252 | A |
| 6262 | - | 6253 | T |
| 6262 | - | 6254 | A |
| 6262 | - | 6255 | A |
| 6262 | - | 6256 | T |
| 6262 | - | 6257 | A |
| 6830 | T | 6826 | C |
| 7149 | T | 7145 | G |
| 7421 | T | 7417 | G |
| 7586 | C | 7582 | - |
| 7587 | T | 7582 | - |
| 7655 | - | 7649 | A |
| 8026 | A | 8021 | C |
| 9646 | A | 9641 | G |
| 9722 | - | 9717 | T |
| 9740 | T | 9736 | A |
| 12218 | T | 12214 | - |
| 12234 | A | 12229 | C |
| 12660 | G | 12655 | A |
| 12813 | G | 12808 | A |
| 13523 | A | 13518 | - |
| 13934 | A | 13928 | G |
| 13960 | - | 13954 | A |
| 15718 | T | 15713 | - |
| 15719 | T | 15713 | - |
| 15845 | A | 15838 | C |
| 16872 | A | 16865 | G |
| 16940 | G | 16933 | T |
| 17774 | T | 17767 | A |
| 18384 | G | 18377 | T |
| 18385 | G | 18378 | C |
| 18386 | A | 18379 | C |
| 18513 | A | 18506 | C |
| 21526 | C | 21519 | A |
| 21625 | T | 21618 | C |
| 24975 | A | 24968 | C |
| 25091 | G | 25084 | A |
| 26079 | A | 26072 | C |
| 26399 | A | 26392 | G |
| 26432 | - | 26425 | A |
| 26432 | - | 26426 | A |
| 26644 | - | 26639 | T |
| 26743 | - | 26739 | A |
| 27097 | T | 27094 | G |
| 27294 | T | 27291 | G |
| 27375 | G | 27372 | T |
| 27714 | - | 27711 | T |
| 29812 | T | 29810 | - |
| 29813 | T | 29810 | - |
| 29814 | A | 29810 | - |
| 29815 | A | 29810 | - |
| 29854 | A | 29848 | C |
| 30114 | G | 30108 | T |
| 30428 | C | 30422 | A |
| 31058 | - | 31052 | A |
| 31058 | - | 31053 | G |
| 31058 | - | 31054 | T |
| 31058 | - | 31055 | A |
| 31058 | - | 31056 | C |
| 31418 | T | 31417 | A |
| 31419 | T | 31418 | A |
| 31426 | T | 31425 | A |
| 32663 | A | 32662 | G |
| 32829 | A | 32828 | - |
| 33345 | G | 33343 | A |
| 34975 | A | 34973 | G |
| 39953 | C | 39951 | T |
| 40811 | C | 40809 | T |
| 42165 | - | 42163 | T |
| 42967 | G | 42966 | A |
| 44330 | T | 44329 | - |
| 45314 | G | 45312 | A |
| 45328 | T | 45326 | G |
| 46461 | T | 46459 | C |
| 46488 | C | 46486 | A |
| 46793 | - | 46791 | A |
| 46946 | T | 46945 | G |
| 46950 | T | 46949 | C |
| 47042 | T | 47041 | - |
| 47045 | T | 47043 | - |
| 47047 | T | 47044 | - |
| 47049 | A | 47045 | - |
| 47052 | A | 47047 | - |
| 47055 | T | 47049 | - |
| 47057 | A | 47050 | - |
| 47060 | T | 47052 | - |
| 47061 | T | 47052 | - |
| 47062 | A | 47052 | - |
| 47063 | T | 47052 | - |
| 47064 | T | 47052 | - |
| 47357 | G | 47344 | A |
| 48022 | T | 48009 | - |
| 49786 | C | 49772 | A |
| 51134 | T | 51120 | G |
| 52770 | T | 52756 | C |
| 53987 | T | 53973 | A |
| 54059 | T | 54045 | G |
| 56980 | C | 56966 | A |
| 57197 | T | 57183 | C |
| 57730 | A | 57716 | C |
| 58841 | T | 58827 | C |
| 58878 | C | 58864 | T |
| 59030 | A | 59016 | G |
| 60348 | T | 60334 | C |
| 60527 | A | 60513 | - |
| 61449 | T | 61434 | G |
| 62588 | C | 62573 | A |
| 62614 | G | 62599 | A |
| 62824 | G | 62809 | A |
| 63189 | G | 63174 | A |
| 65075 | C | 65060 | - |
| 65076 | T | 65060 | - |
| 65077 | A | 65060 | - |
| 65078 | T | 65060 | - |
| 65079 | A | 65060 | - |
| 65080 | A | 65060 | - |
| 65081 | T | 65060 | - |
| 65082 | G | 65060 | - |
| 65083 | A | 65060 | - |
| 65084 | T | 65060 | - |
| 65085 | T | 65060 | - |
| 65224 | C | 65198 | A |
| 67136 | T | 67110 | A |
| 67137 | A | 67111 | T |
| 67641 | T | 67615 | G |
| 67682 | C | 67656 | - |
| 67683 | T | 67656 | - |
| 67684 | T | 67656 | - |
| 67685 | A | 67656 | - |
| 67686 | A | 67656 | - |
| 67687 | T | 67656 | - |
| 68512 | T | 68480 | C |
| 68513 | G | 68481 | A |
| 68514 | T | 68482 | C |
| 68515 | G | 68483 | A |
| 68891 | - | 68859 | T |
| 68891 | - | 68860 | T |
| 68891 | - | 68861 | T |
| 68891 | - | 68862 | A |
| 68891 | - | 68863 | T |
| 68891 | - | 68864 | T |
| 68891 | - | 68865 | C |
| 68891 | - | 68866 | G |
| 68891 | - | 68867 | A |
| 69091 | A | 69068 | C |
| 69502 | - | 69479 | A |
| 70779 | - | 70757 | T |
| 70779 | - | 70758 | T |
| 73536 | A | 73516 | C |
| 74033 | C | 74013 | A |
| 74034 | T | 74014 | G |
| 74329 | A | 74309 | - |
| 75829 | C | 75808 | A |
| 76351 | A | 76330 | - |
| 76352 | T | 76330 | - |
| 76353 | T | 76330 | - |
| 76354 | A | 76330 | - |
| 76355 | T | 76330 | - |
| 77970 | C | 77944 | T |
| 78717 | G | 78691 | T |
| 79013 | A | 78987 | C |
| 80410 | A | 80384 | C |
| 80447 | C | 80421 | T |
| 81544 | - | 81518 | A |
| 81544 | - | 81519 | T |
| 81544 | - | 81520 | A |
| 81544 | - | 81521 | A |
| 81544 | - | 81522 | T |
| 81544 | - | 81523 | A |
| 81544 | - | 81524 | T |
| 81544 | - | 81525 | T |
| 81544 | - | 81526 | T |
| 81544 | - | 81527 | A |
| 81544 | - | 81528 | T |
| 81544 | - | 81529 | A |
| 81544 | - | 81530 | T |
| 82826 | T | 82813 | A |
| 89971 | T | 89958 | G |
| 93067 | G | 93054 | T |
| 93068 | G | 93055 | C |
| 93069 | A | 93056 | C |
| 94410 | C | 94397 | T |
| 103300 | A | 103287 | G |
| 108499 | C | 108486 | A |
| 108584 | A | 108571 | C |
| 108852 | T | 108839 | A |
| 109007 | T | 108994 | G |
| 109115 | C | 109102 | T |
| 109927 | T | 109914 | A |
| 110971 | T | 110958 | G |
| 110982 | T | 110969 | C |
| 111255 | - | 111242 | A |
| 111255 | - | 111243 | A |
| 111255 | - | 111244 | A |
| 111255 | - | 111245 | A |
| 111255 | - | 111246 | A |
| 111255 | - | 111247 | A |
| 112215 | C | 112208 | T |
| 112265 | T | 112258 | - |
| 112266 | G | 112258 | - |
| 114132 | - | 114123 | G |
| 114133 | T | 114125 | A |
| 114135 | C | 114127 | - |
| 114142 | C | 114133 | T |
| 114210 | A | 114201 | G |
| 116414 | T | 116405 | C |
| 117217 | T | 117208 | C |
| 118534 | A | 118525 | - |
| 121769 | A | 121759 | C |
| 122130 | C | 122120 | A |
| 122144 | A | 122134 | - |
| 122179 | T | 122168 | G |
| 122313 | C | 122302 | A |
| 122355 | G | 122344 | T |
| 122356 | A | 122345 | T |
| 122357 | A | 122346 | T |
| 122358 | A | 122347 | T |
| 122359 | A | 122348 | C |
| 122363 | G | 122352 | T |
| 122414 | T | 122403 | - |
| 122716 | T | 122704 | G |
| 122943 | T | 122931 | C |
| 123880 | T | 123868 | G |
| 123969 | G | 123957 | A |
| 124673 | C | 124661 | A |
| 124782 | G | 124770 | A |
| 125147 | T | 125135 | C |
| 125558 | T | 125546 | C |
| 125792 | C | 125780 | A |
| 125877 | G | 125865 | A |
| 125941 | T | 125929 | G |
| 125984 | T | 125972 | C |
| 126145 | A | 126133 | G |
| 126491 | T | 126479 | A |
| 126625 | T | 126613 | G |
| 126626 | C | 126614 | A |
| 127122 | T | 127110 | G |
| 127207 | G | 127195 | T |
| 132406 | T | 132394 | C |
| 141296 | G | 141284 | A |
| 142637 | T | 142625 | G |
| 142638 | C | 142626 | G |
| 142639 | C | 142627 | A |
| 145735 | A | 145723 | C |


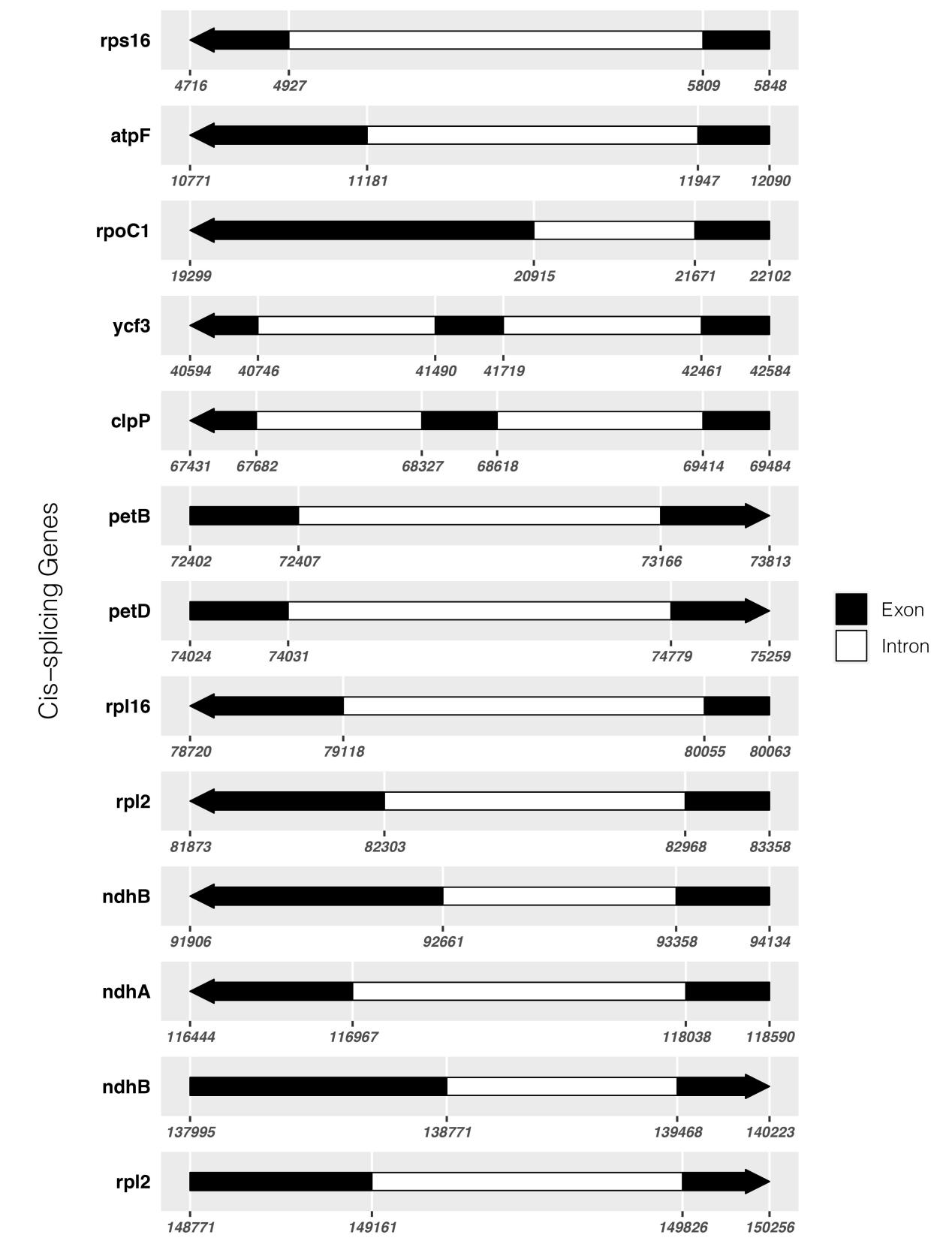


# Figure S1. Schematic presentation of the structure of cis-splicing genes of the chloroplast genome of *I. japonica*. The white area represents Intron, and the black area represents the exon. The arrow shows the sense direction of the genes.


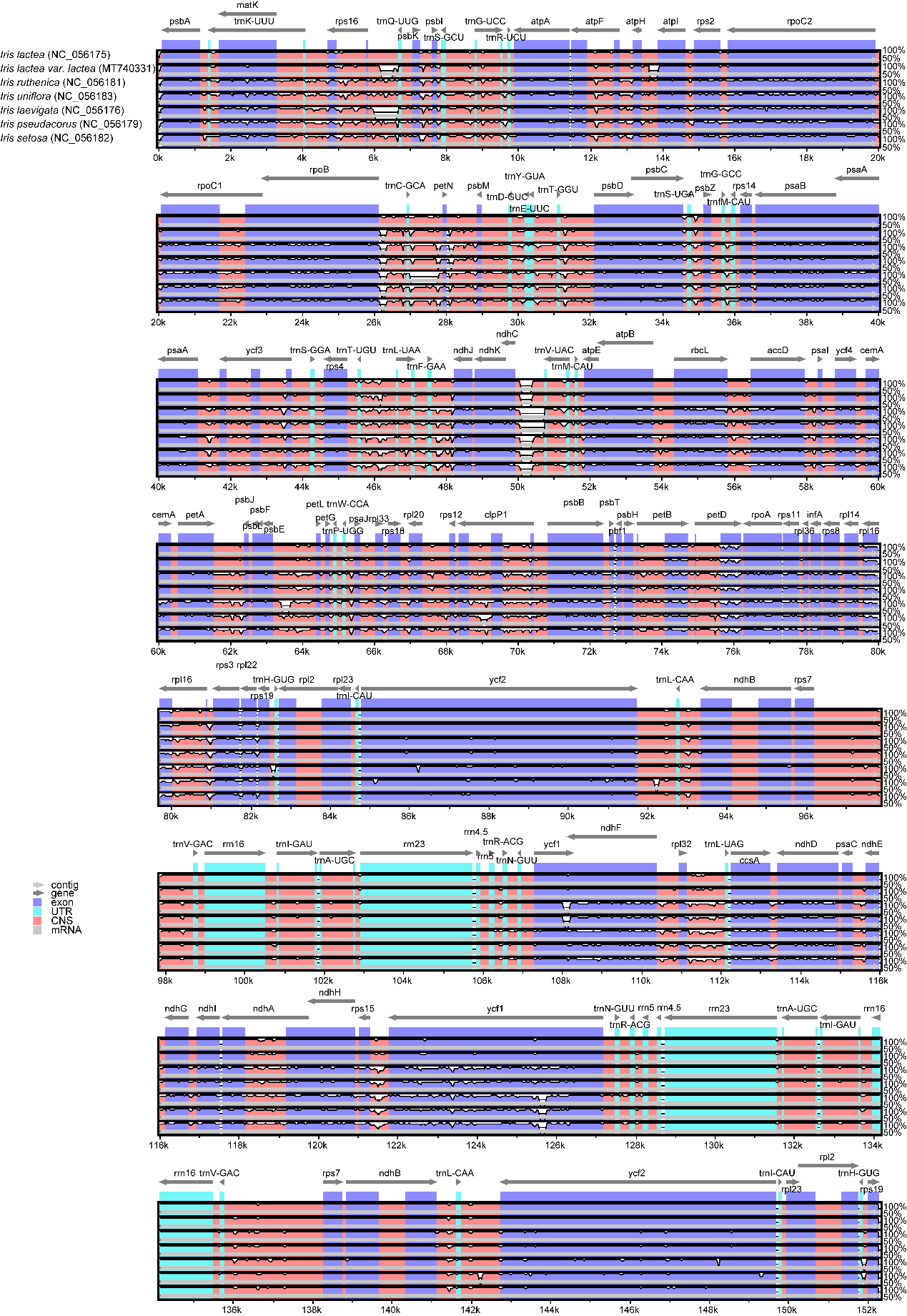


# Figure S2. Sequence alignment of seven chloroplast genome of Iris species using mVISTA and chloroplast genome of *I. lactea* (NC_056175) as reference. The top arrow shows transcription direction, blue colour indicates protein coding regions, pink colour shows non-coding sequences and light green indicates tRNAs and rRNAs. The x-axis represents the coordinates in the cp genome while y- axis represents percentage identity within 50–100%.


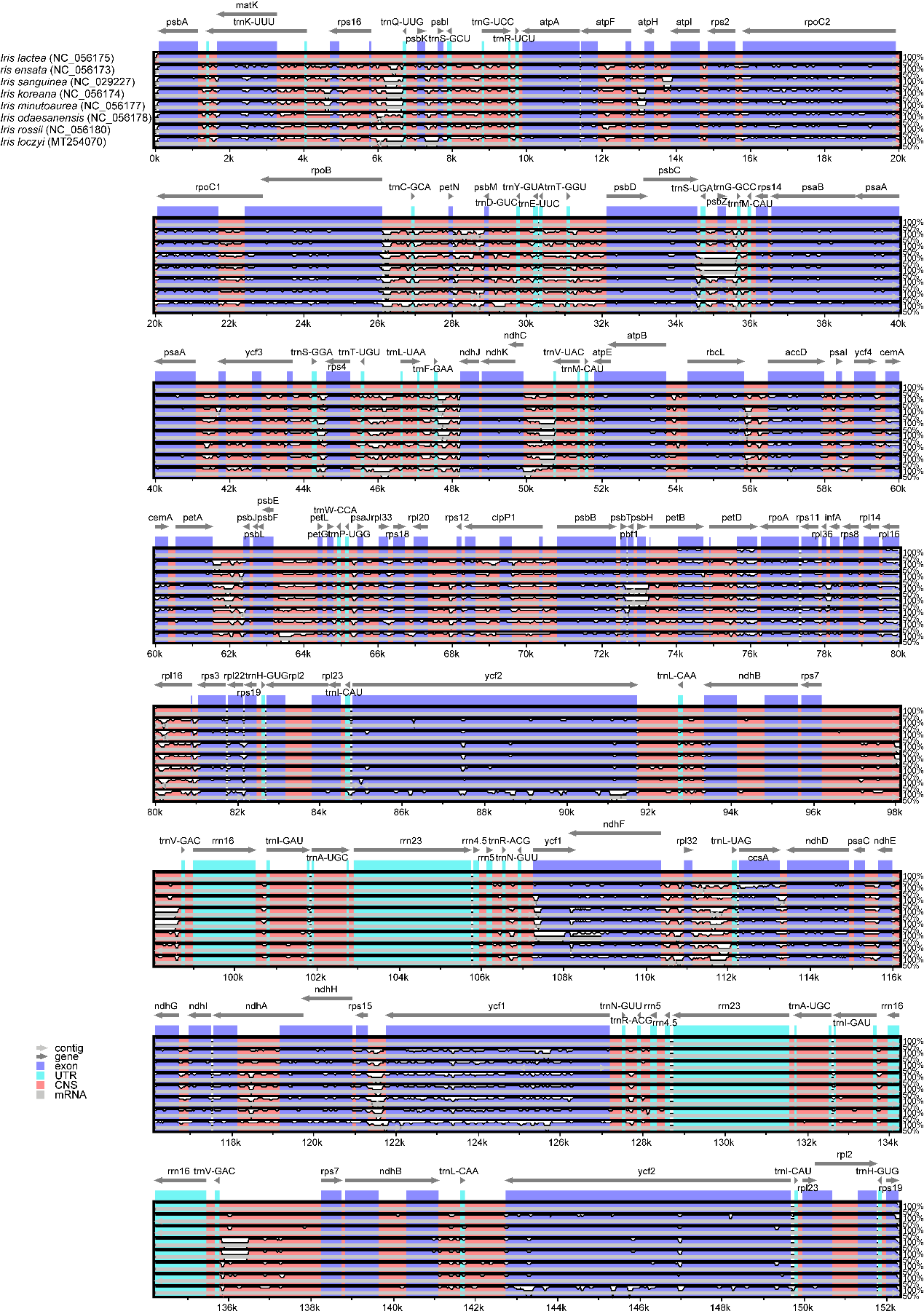


# Figure S3. Sequence alignment of seven chloroplast genome of Iris species using mVISTA and chloroplast genome of *I. lactea* (NC_056175) as reference. The top arrow shows transcription direction, blue colour indicates protein coding regions, pink colour shows non-coding sequences and light green indicates tRNAs and rRNAs. The x-axis represents the coordinates in the cp genome while y- axis represents percentage identity within 50–100%.


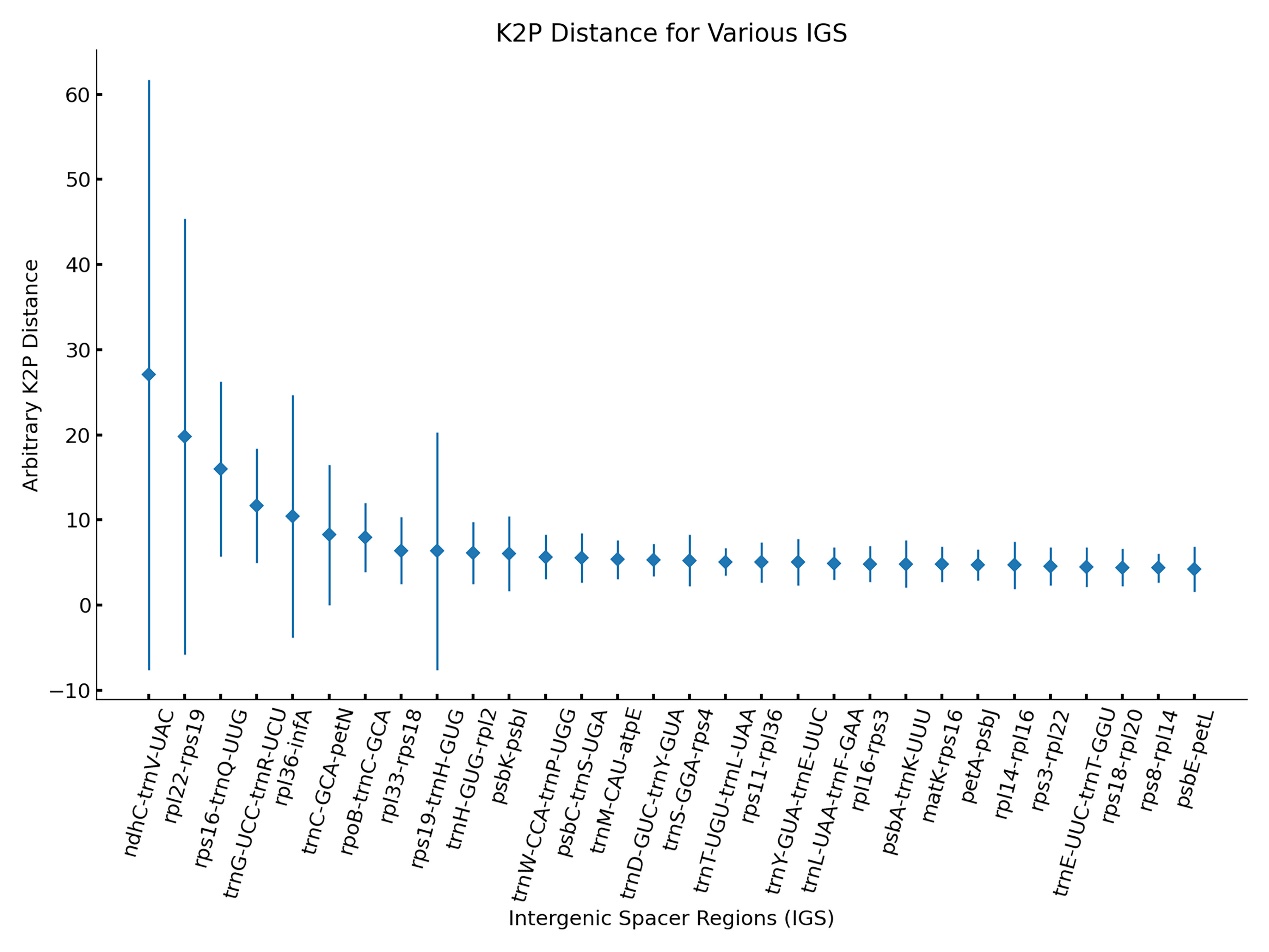


# Figure S4. Average K2p distances among the intergenic spacer regions from the 20 *Iris* species: *I. lactea*, *I. lactea* var *lactea*, *I. ruthenica*, *I. uniflora*, *I. laevigata*, *I. pseudacorus*, *I. setosa*, *I. ensata*, *I. sanguinea*, *I. koreana*, *I. minutoaurea*, *I. odaesanensis*, *I. rossii*, *I. loczyi*, *I. speculatrix*, *I. missouriensis*, *I. tectorum*, *I. japonica*, *I. domestica*, and *I. dichotoma*. The K2p distances were calculated for the corresponding IGS regions pairwisely. The black dots represent the average K2p distances for the corresponding IGS regions.
